# Outstanding Sorption of Copper (II) Ions on Porous Phenothiazine-Imine-Chitosan Materials

**DOI:** 10.3390/gels9020134

**Published:** 2023-02-04

**Authors:** Andrei Bejan, Luminita Marin

**Affiliations:** “Petru Poni” Institute of Macromolecular Chemistry, Gr. Ghica Voda Alley, 41A, 700487 Iasi, Romania

**Keywords:** chitosan, phenothiazine, copper retention

## Abstract

The aim of this work was to investigate the ability of a solid-state material, prepared by crosslinking chitosan with a phenothiazine-based aldehyde, to remove copper (II) ions from aqueous solutions, in a fast and selective manner. The metal uptake experiments, including the retention, sensibility, and selectivity against eight different metal ions, were realized via batch adsorption studies. The capacity of the material to retain copper (II) ions was investigated by spectrophotometric measurements, using poly(ethyleneimine) complexation agent, which allowed detection in a concentration range of 5–500 µM. The forces driving the copper sorption were monitored using various methods, such as FTIR spectroscopy, X-ray diffraction, SEM-EDAX technique, and optical polarized microscopy, and the adsorption kinetics were assessed by fitting the in vitro sorption data on different mathematical models. The phenothiazine-imine-chitosan material proved high ability to recover copper from aqueous media, reaching a maximum retention capacity of 4.394 g Cu (II)/g adsorbent when using a 0.5 M copper solution, which is an outstanding value compared to other chitosan-based materials reported in the literature to this date. It was concluded that the high ability of the studied xerogel to retain Cu (II) ions was the result of both physio- and chemo-sorption processes. This particular behavior was favored on one hand by the porous nature of the material and on the other hand by the presence of amine, hydroxyl, imine, and amide groups with the role of copper ligands.

## 1. Introduction

We live in a world that faces major global problems, including the continuous degradation of the environment with its unwanted component, pollution, which, through its omnipresence, has a dramatic influence especially on living organisms. Of the main polluting agents, the presence of heavy metals in the environment has gained quite a lot of attention due to their increased release in water sources and also due to their toxicity [1,2].

Among these toxic heavy metals, along with the development of the agricultural and industrial sectors, copper has become one of the chemical elements most widely used nowadays [3]. However, it is not biodegradable, it is poisonous, and at low concentrations it can be easily accumulated in living organisms, including human beings [4,5].

Because of these severe consequences, there is a continuous demand for the development of new decontamination techniques and materials. Among the numerous methods known and used up to now, the adsorption technique seems to be the most widely applied, due to its low cost and simple operation process [6]. In recent years, one of the newest developments in terms of heavy metal removal was biosorption, which employs the use of natural-based polymers as adsorbents [7,8,9]. Particularly, chitosan, a derivative of chitin (which is a naturally occurring polysaccharide from crustacean and fungal biomass), proved to be the most worthy candidate, both due to the abundance of raw material and implicitly the low costs of processing and obtaining, and also due to the fact that it has been found to be capable of chemically or physically adsorbing various heavy metal ions, copper included [10,11,12,13,14,15]. Various approaches were considered, mainly by combining chitosan with other components and manufacturing them as materials meant to increase copper adsorption ability [6,16]. In Table 1 below, we summarize some of the most notable results in terms of copper ion adsorption on chitosan-based materials.

Our studies indicated that the combination of chitosan with phenothiazine via imine bonds lead to hydrogels and corresponding xerogels with high ability to recover heavy metals such as mercury [13,29]. Herein, we extended the studies to the utilization of a chitosan-phenothiazine-based material as an adsorbent for the copper ions from aqueous samples. A series of batch adsorption experiments were conducted under controlled experimental conditions, and the results of the investigation are discussed. 

## 2. Results and Discussions

### 2.1. Design and Preparation of Xerogel

**Scheme 1:**
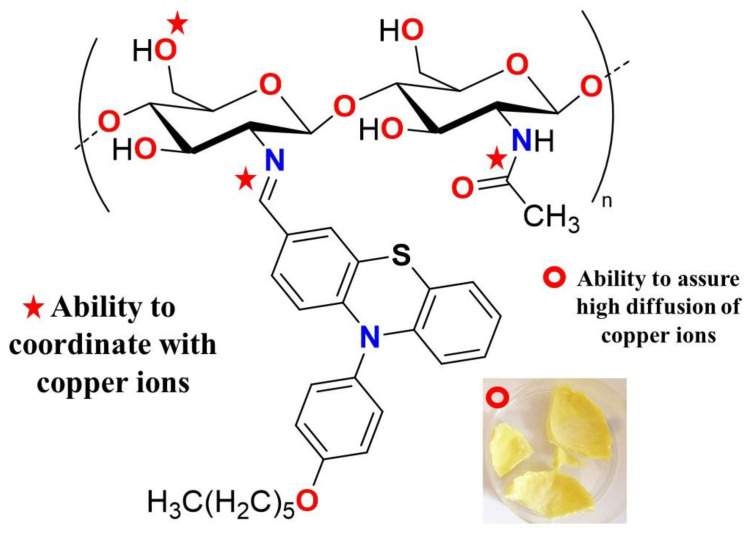
Rational design of the material.

A xerogel based on chitosan and phenothiazine heterocycle has been prepared following a procedure reported elsewhere [30], and its ability to recover copper was investigated by batch method. From chemical point of view, the xerogel consists of phenothiazine units linked to chitosan via imine bonds, and all three components, chitosan, imine bonds and phenothiazine, haveg good complexation ability for copper [31,32,33,34,35,36,37]. The combination of chitosan with phenothiazine via imine bonds leads to hydrogels, which by lyophilization formed the corresponding xerogels, with a porous morphology. Thus, combining these three chemical building blocks into a porous material creates good premises for increasing the copper sorption ability (Scheme 1). 

### 2.2. Copper Removal from Controlled Contaminated Drinking Water Maximum Copper Retention Assay

**Figure 1:**
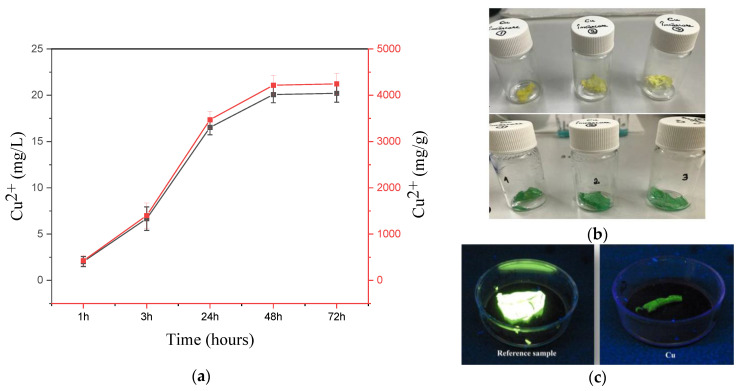
(**a**) Copper retention capacity (mg/L and mg/g) over time, from 0.5 M solutions; (**b**) Images of the xerogel before/after copper sorption under natural light; (**c**) Images of the xerogel before/after copper sorption under UV light.

The ability of the studied xerogel to recover copper from wastewater was firstly assessed in concentrated copper aqueous solution (0.5 M, controlled contaminated). The copper sorption kinetics of the xerogel showed that sorption equilibrium was been reached after 48 h (Figure 1a). The sorption was rapid in the first 24 h and slow in the next 24 h, reaching a maximum copper ion retention capacity of 4394 mg Cu (II)/g xerogel with a good retention efficiency of 79%, value comparable to that obtained in the case of a standard solution made with ultrapure water. This is an outstanding result, compared to the data reported in the literature up to now, on different chitosan-based materials (Table 1) [6]. After the experiment, the xerogel transformed into a green transparent semisolid material with no obvious salt deposits (Figure 1b). Moreover, the high ability of the xerogel to emit yellow light was almost quenched, only slight green light emission remaining (Figure 1c).

**Figure 2:**
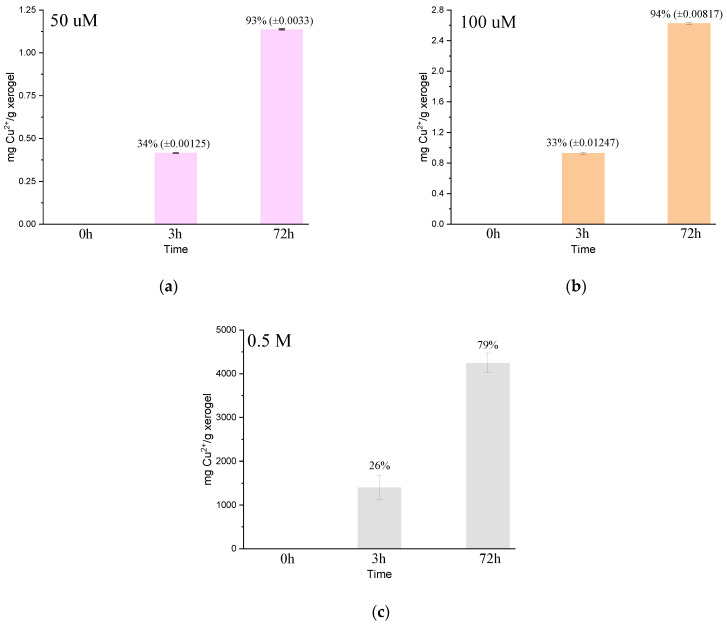
The sorption behavior (retention capacity and efficiency) from three controlled-contaminated water samples of (**a**) 50, (**b**) 100 µM, and (**c**) 0.5 M.

Considering this promising result, the next step of the study was the simulation of three batches of wastewater, by contaminating three drinking water samples with copper ions (Figure 2a–c). In order to test the ability of the xerogel to remove the copper ions even at low concentrations, it was chosen to work at very low concentrations of 50 µM and 100 µM, respectively. In addition, working that low in terms of concentration, we managed to test the sensitivity of the xerogel towards copper ions.

As can be seen in Figure 2a,b, even at these very low concentrations, the xerogel exhibited good retention capacities, being able to uptake copper ions up to 1.137 mg/g xerogel, and 2.623 mg/g xerogel, respectively, with a retention efficiency of 93-94% (see Equation (4) from the Section 4).

#### Selectivity Assay towards Copper Ions

The selectivity studies were performed applying a batch sorption experiment protocol, in which nine different metal ions were used, as follows: copper, sodium, cadmium, zinc, manganese, nickel, cobalt, lead, and barium. After the batch sorption experiments, the samples were further processed and analyzed with the help of the SEM-EDAX technique. The analysis was performed on the loaded samples, investigating a minimum of five areas of the xerogels. The given data were provided as the mass ratio (W%) of the copper atoms to the rest of the atoms found in the chemical composition of the xerogel.

**Figure 3:**
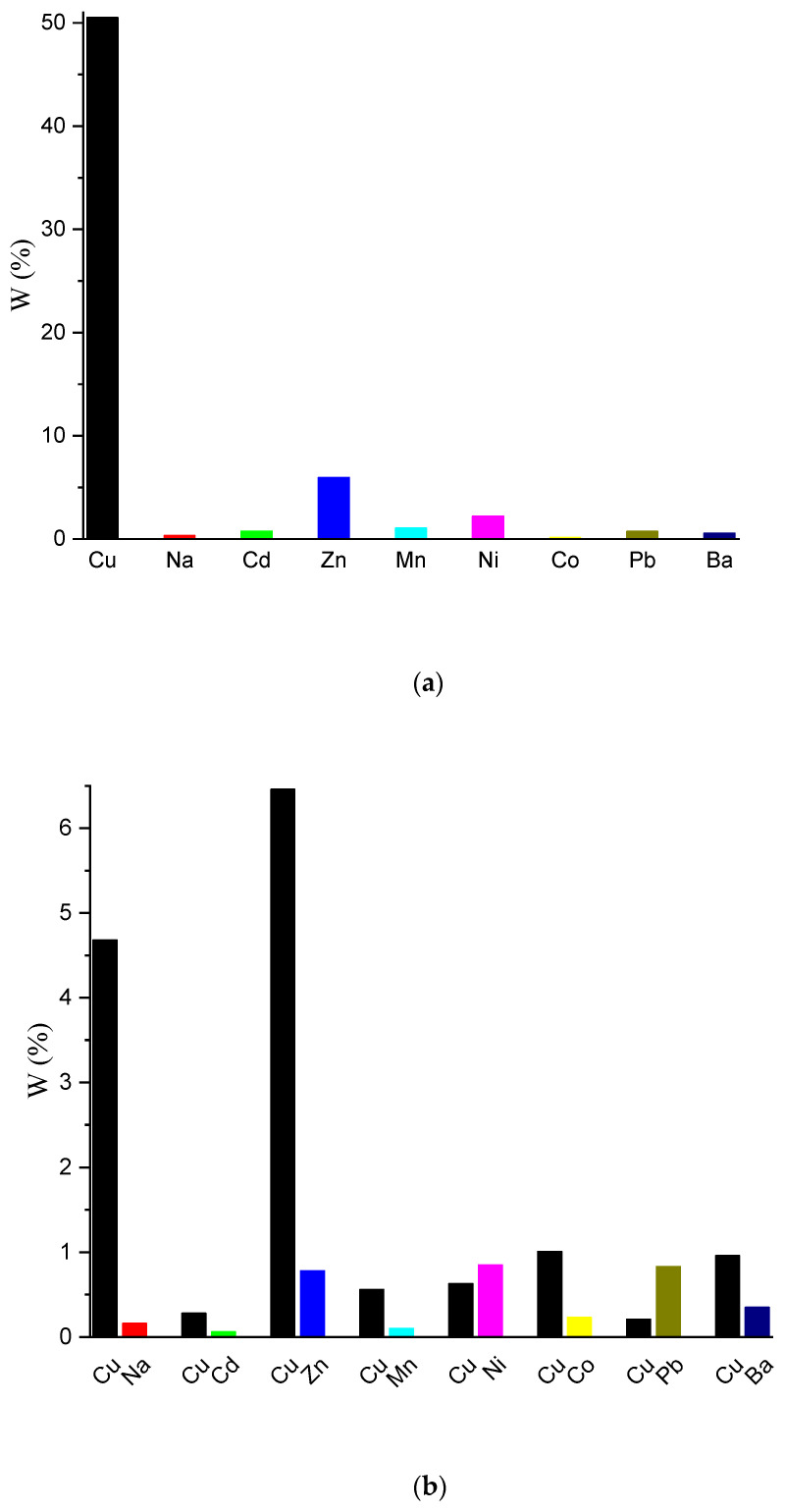
Selectivity analysis given by SEM-EDAX technique: the mass ratio of metal ions into xerogel when the experiment was performed with (**a**) individual solutions of metal salts and (**b**) mixed binary solutions of copper salt with different metals.

In terms of selectivity, as can be seen in Figure 3, the best result was obtained in the case of the copper ions sorption, which is in a good agreement with the experimental data exposed so far, proving the ability of the material to interact preferentially with the copper ions. Nonetheless, there were two exceptions, in the case of nickel and lead ions, due to their mutual interference behavior with the copper ions [38,39].

### 2.3. Copper Ion Interactions with the Xerogel Matrix 

In order to establish the driving forces that guided the copper retention into the xerogel, an exhaustive physico-chemical analysis of the xerogels before and after retention experiments was undertaken. 

#### 2.3.1. FTIR Characterization

**Figure 4:**
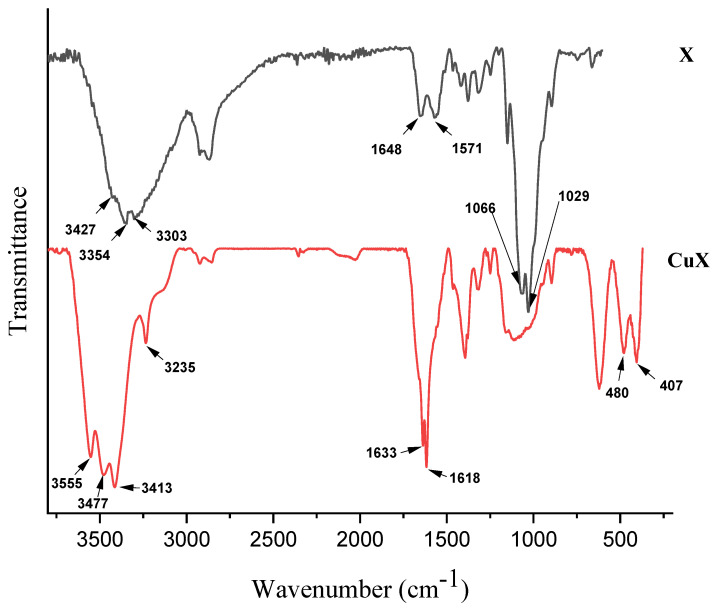
Comparative FTIR spectra (reference xerogel vs. xerogel in the presence of Cu (II) ions).

First clues regarding the chemical anchoring of the copper ions into the xerogel were obtained when analyzing the comparative FTIR spectral characteristics of the samples, without/with Cu^2+^ ions (Figure 4). On the first look, the FTIR spectra of CuX showed drastic alterations, indicating the chemical bonding of copper ions into the chitosan-based matrix. Thus, a first modification could be observed in the 3600–3200 cm^−1^ spectral domain, corresponding to the amine, hydroxyl, and their intra- and inter-molecular hydrogen bond formation [30,40,41,42]. The broad band with overlapped maxima at 3427, 3354, and 3303 cm^−1^ transformed into one with distinct sharp maxima, positioned at significantly shifted wavenumbers, at 3555, 3477, 3413, and 3235 cm^−1^, suggesting the involvement of amine and hydroxyl groups in the formation of coordination bonds with the copper ions [43]. This attribution was supported by the modification of the vibration bands specific to the hydroxyl groups of the chitosan from 1066, 1029 cm^−1^, which transformed into a broad band of lower intensity in the 1171–988 cm^−1^ range, in agreement with the modification of the hydrogen bond environment that generated them [44,45]. Moreover, the scissoring and bending vibration bands of the C-H bonds of the -CH_2_- units shifted from 1413 cm^−1^ to 1395 cm^−1^, supporting once more the modification in the environment of -CH_2_OH groups due to the presence of copper ions [44].

Another suggestive behavior was noticed in the case of the imine vibration band (1648 cm^−1^), which was also shifted to a lower wavenumber (1633 cm^−1^), being associated with the involvement of the nitrogen atom in the complexation of the copper ions [46]. Similarly, the amide vibration band was shifted from 1571 to 1618 cm^−1^, suggesting its involvement in the copper complexation too.

A very clear modification of the CuX spectra was observed in the low-frequency region, with the appearance of two distinct vibration bands (480, 407 cm^−1^), which are characteristic for metal-carbon bonds and could be assigned to a dative bond created between copper ions and the nitrogen atom involved in the imine linkage [47].

#### 2.3.2. Wide-Angle X-ray Diffraction

**Figure 5:**
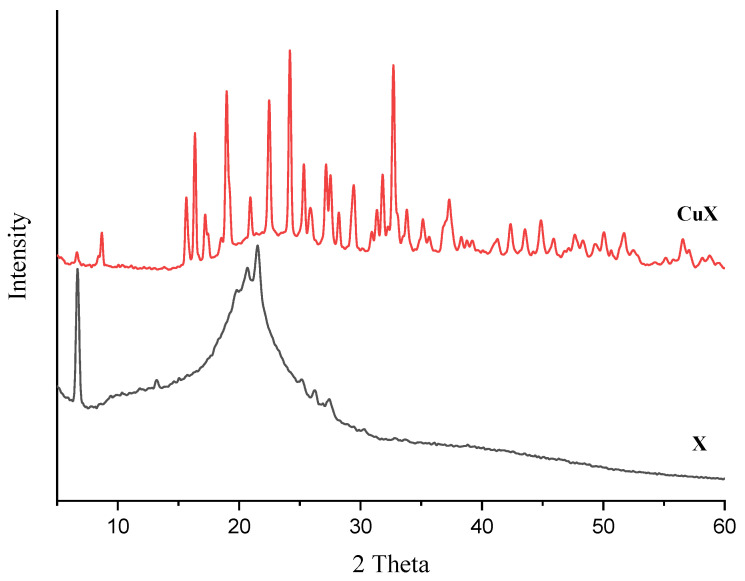
X-ray diffractograms of the X and CuX sample.

In order to gain further information regarding the anchoring of the copper into the xerogel network, the X-ray diffractograms of the CuX and X samples were comparatively investigated (Figure 5).

The reference xerogel (X) diffractogram presented a sharp reflection at 6.6° and a broader one with maxima at 20° and 21.4°, characteristic of the chitosan hydrogelation with the phenothiazine-based aldehyde, by forming layered clusters that played the role of crosslinking nodes [29,30].

Compared to the reference xerogel, the diffractogram of the CuX sample showed a larger number of sharp reflection peaks, indicating the presence of a distinct crystalline phase [48]. Compared to the crystallographic cards for copper (Copper__R061078, from RRUFF Database) and copper salt used in the experiment (Cu(SO_4_)·5H_2_O_R060102, from RRUFF Database), the reflections in the diffractograms of CuX samples were shifted to lower/higher angle. This indicates that the crystallization pattern was affected by the interaction of the cupper with xerogel matrix, either by chemical or physical forces, confirming once more the successful anchoring of the copper ions into the xerogel matrix, as FTIR spectra suggested. 

#### 2.3.3. Polarized Light Optical Microscopy (POM) and Scanning Electron Microscopy (SEM)

**Figure 6:**
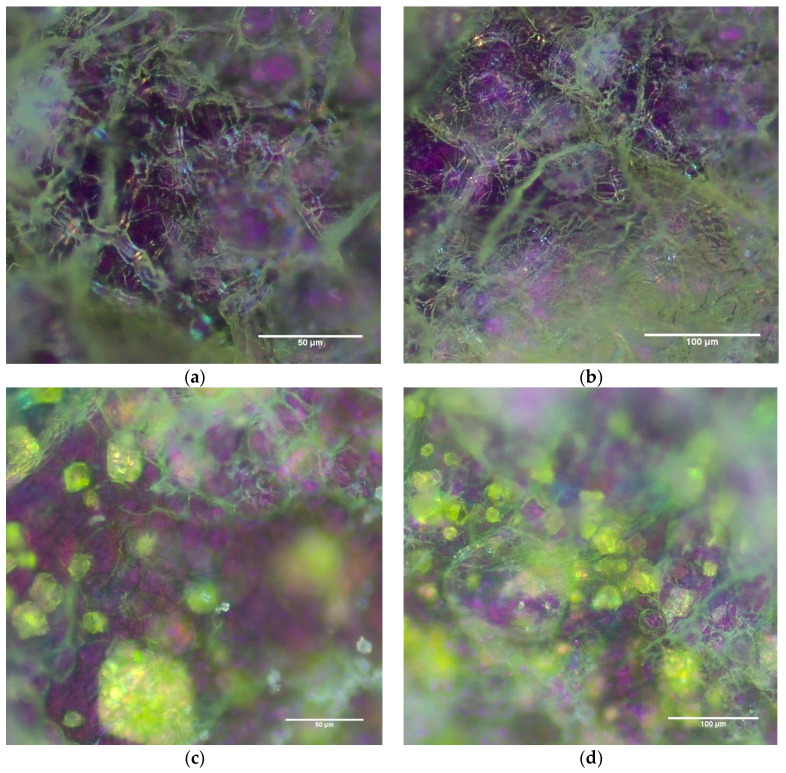
Representative POM images of X reference xerogel (**a**,**b**) and CuX sample (**c**,**d**).

The supramolecular ordering of the samples given by the X-ray diffraction technique was also confirmed by the polarized light microscopy images acquired at different magnification scales. As can be seen in Figure 6, both samples (reference xerogel- Figure 6a,b and CuX- Figure 6c,d) presented a birefringent porous microstructure. Interestingly enough, in addition to the birefringent character of the reference xerogel [30], in the case of the CuX sample strong birefringent clusters inside the porous material were evidenced, suggesting some supramolecular entities generated by the presence of copper ions. However, no geometric forms characteristic for the crystals of copper salts or copper were present, confirming that the copper ions interacted with the xerogel matrix leading to a new supramolecular ordering pattern. 

**Figure 7:**
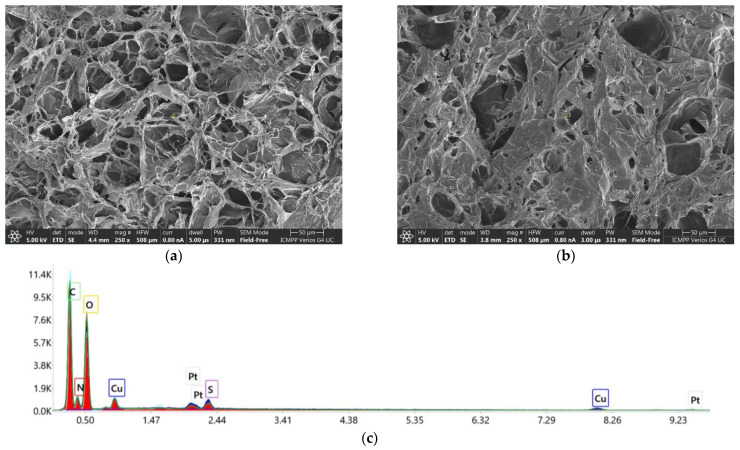
Reference X (**a**), CuX (**b**) SEM micrographs and EDAX plot for the CuX sample (**c**).

The morphology of both samples was investigated by scanning electron microscopy. As can be seen in Figure 7a, the reference xerogel presented a sponge-like morphology with interconnected pores with diameter around 47 µm and thin walls around 2.85 µm) [30].

The sorption of copper ions impacted a drastic modification of the xerogel morphology. Although the pore diameters remained in the same dimension range, at the level of the pore walls a significant thickening was noticed, demonstrating an obvious mass transfer of copper ions into the xerogel matrix, attributed to a sorption process [19].

The efficient encapsulation of the copper ions into the porous sample was also confirmed by EDAX analysis, which indicated a 50% (±2.6) mass percent of metal.

### 2.4. Adsorption Kinetics

**Figure 8:**
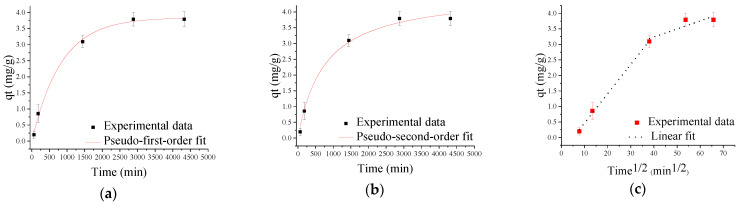
Fitting of the nonlinear kinetic models for the sorption of copper ions on the reference X xerogel: pseudo-first-order (**a**), pseudo-second-order (**b**), and Weber–Morris (**c**) fit.

In order to understand the characteristics of the sorption phenomenon that took place when using the chitosan-phenothiazine based xerogel and also to identify the dynamics of this adsorption process, three kinetic models were applied, as follows: pseudo-first-order, pseudo-second-order, and Weber–Morris’s intraparticle diffusion [49]. In Figure 8 the fitting of the nonlinear kinetic models can be seen.

As can be seen from the kinetic parameters (Table 2), the experimental data were well fitted when using the nonlinear pseudo-first-order kinetic model: the equilibrium adsorption capacity value (q_e, exp_) is quite close to the experimental one (q_e, calc_); the correlation coefficient (R^2^) and the Chi-square test (χ^2^) is the highest and the lowest, respectively. All these indicate that physisorption is the predominant mechanism involved in copper retention into the chitosan-phenothiazine xerogels. These findings are also in good agreement with the literature involving the adsorption kinetics of some chitosan-based adsorbents [50,51].

Nonetheless, corroborating the information extracted from FTIR, WAXD, and SEM and also from the nonlinear pseudo-second-order kinetic model parameters, it is obvious that the chemisorption phenomenon was also involved (Table 2).

The intraparticle diffusion model, fitted with the experimental data, revealed an adsorption process with two distinct stages (Figure 8c, Table 2). Considering the other experimental data, the first stage was attributed to the migration of the copper ions from solution to xerogel and into its pores (surface diffusion), while the second linear step was assigned to the adsorption of the copper into and onto the pore walls [52,53].

### 2.5. Adsorption Isotherms

**Figure 9:**
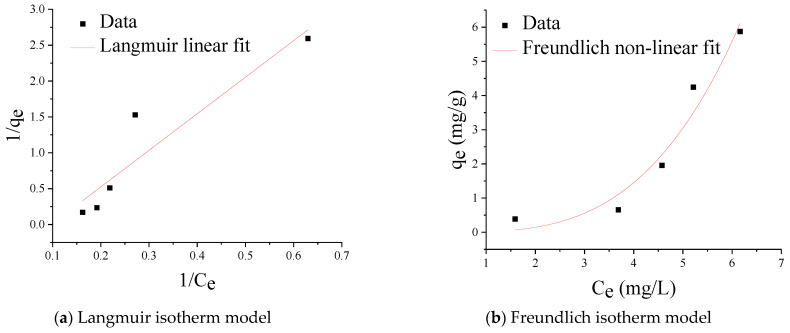

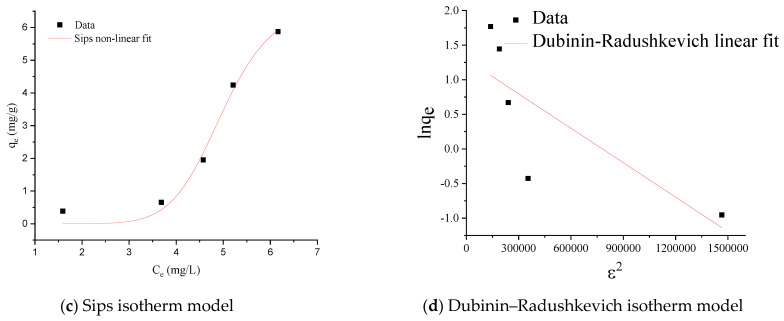
The fitting of copper retention data on different isotherm models.

The experimental data, collected using the batch sorption technique, were further examined by four isotherm models: Langmuir (linear model), Freundlich (nonlinear model), Sips (nonlinear model), and Dubinin–Radushkevich (nonlinear model) as well (Figure 9a–d). In Table 3 the relevant parameters for the above-mentioned isotherm models are presented, calculated based on the experimental data.

The feasibility of the adsorption process into the samples was firstly confirmed by the equilibrium parameter value (R_L_ = 0.21) obtained when applying the Langmuir model. According to the literature, an adsorption process is considered favorable when 0 < R_L_ < 1 [49,52,53,54,55].

Analyzing the values obtained for all four models, it was concluded that the best fit was in the case of the Sips nonlinear isotherm (Figure 9c, Table 3). Some additional information was also extracted from the Dubinin–Radushkevich isotherm model parameters (Figure 9d, Table 3). The sorption free energy value (E, kJ/mol) calculated with this model confirmed once more the fact that in our case, the dominant adsorption mechanism was based on physisorption [49]. A plausible explanation of the fact that there are such differences between the values obtained by modeling the adsorption isotherms and the effective value determined experimentally could lie in what concerns the morphology of the material. Unlike the mathematical models used that can predict monolayer or interface adsorption phenomena, the adsorption of our material is enhanced by its porous nature, which generates a high active surface, allowing copper ions to easily penetrate the matrix, embedding them through physical processes.

## 3. Conclusions

A porous xerogel sample, based on chitosan and phenothiazine bonded via imine units, was prepared and investigated for its ability to recover copper from wastewater. This chemical combination proved high ability to retain copper ions due to its ability to develop intermolecular forces and thus to anchor the copper ions, as was proved by FTIR, X-ray, POM, and SEM measurements. Batch experiment indicated a high ability of this material to selectively retain copper, reaching 4394 mg Cu^2+^/g xerogel from 0.5 M aqueous solutions, and a 94% removal efficiency from 50 µM controlled-contaminated solution. Modeling of the kinetic studies and the adsorption isotherms by fitting the experimental data on mathematical models indicated that the high retention was the result of combined chemisorption and physisorption processes, the last one being dominant. All in all, the remarkable sorption capacity recommends the studied xerogel as a promising adsorbent for the retention of copper ions in contaminated water specimens.

## 4. Materials and Methods

### 4.1. Materials

Low molecular weight chitosan (263 kDa, 83% deacetylation degree) was purchased from Sigma-Aldrich Co. (USA) and used as received. 10-(-4-Hexyloxy-phenyl)-10H-phenothiazine-3-carbaldehyde was obtained in our laboratory [56]. Inorganic salts: copper sulphate (CuSO_4_ · 5H_2_O, 98%), sodium sulphate (Na_2_SO_4_ anhydrous, 99%), cadmium sulphate (CdSO_4_ · 8/3H_2_O), zinc sulphate (ZnSO_4_ · 7H_2_O, 99%), manganese sulphate (MnSO_4_ · H_2_O, 99%), nickel sulphate (NiSO_4_ · 6H_2_O, 98%), cobalt sulphate (CoSO_4_ · 7H_2_O, 99%), lead sulphate (PbSO_4_, 98%), and barium sulphate (BaSO_4_, 99%) were purchased from Sigma-Aldrich and used as received. Polyethyleneimine (branched, M.W. 10,000, 99%) was purchased from Alfa Aesar and used without further modifications. 

### 4.2. Xerogel (X) Obtaining

The xerogel was obtained using a protocol from our previous work [30]. Briefly, 60 mg of chitosan was dissolved in 0.7% acetic acid in order to form a 2% solution. After that, 5.9 mg 10-(4-hexyloxy-phenyl)-10H-phenothiazine-3-carbaldehyde [56] dissolved in 0.86% DMSO was slowly added under magnetic stirring, until a viscous opaque solution was obtained. This opaque solution was further transformed into a homogenous hydrogel (clear semisolid) by adding 1 mL of acetone. By casting the hydrogel into a Petri dish, a free-standing hydrogel film was obtained, with a thickness of around 2 mm. The hydrogel film was washed with ethanol by successive immersions in ethanol (3×) in order to remove the DMSO, and then it was rehydrated by successive immersions in ultrapure water (3×). Finally, the film was cut into 1 × 1 cm^2^ pieces and lyophilized, giving the corresponding xerogel, coded X.

### 4.3. Methods and Equipment

#### 4.3.1. Equipment

The reference X xerogel was obtained by freezing the corresponding hydrogel in liquid nitrogen and then lyophilizing it using a LABCONCO FreeZone Freeze Dry System equipment, at −50 °C and 1.510 mbar for 24 h.

The Fourier-transform infrared (FTIR) spectra were recorded with a FT-IR Bruker Vertex 70 Spectrophotometer, using KBr pellets, and processed with the help of Origin PRO 2020 software (OriginLab Corporation, Northampton, MA, USA).

The X-ray diffraction was performed with a Benchtop Miniflex 600 Rigaku diffractometer (Tokyo, Japan), from 2 to 60°, registered with 0.01 step and 3°/min speed, on the xerogel pellets.

Micrographs of the samples were acquired with a Polarized Optical Microscope (Zeiss Axio Imager.A2m, camera Axiocam 208 cc (Wetzlar, Germany)), and using a scanning electron microscope Quanta 200 device equipped with an energy dispersive X-ray module (EDX), at accelerated electron energy of 20 keV. For data accuracy, the EDX measurements were taken at five different points, and an average value of the copper mass in samples was considered. The statistical measurements for the xerogel pores and walls diameter were taken using the ImageJ software.

The spectrophotometric determinations used for generating the calibration curve and the retention capacity of xerogel towards copper ions and also for the establishing kinetic parameters and adsorption isotherms were carried out on an Agilent Cary 60 UV-Vis spectrophotometer (Agilent Technologies, Inc. Headquarters, Santa Clara, CA, USA).

All the solutions were prepared using ultrapure water (obtained with the help of a TKA GenPure UF/UV 08.2204 apparatus, Thermo Scientific, Waltham, MA, USA). For the controlled-contaminated water samples, we used drinking water from our available water source.

#### 4.3.2. Methods

All the *copper uptake experiments* involved in this study were performed via batch adsorption assays, as follows: square pieces of xerogel (X) of 1 cm^2^ of known weight were immersed into vials containing 5 mL aqueous copper solutions (50 µM, 100 µM, 0.5 M) and maintained into an incubator, at 25 °C and 25 rpm, for a period of 72 h. At certain moments, 30 µL of the supernatant was extracted from the vials and properly diluted in order to be analyzed using the spectrophotometric technique by fitting the absorbance on a calibration curve. 

The *selectivity assay* was performed in a similar manner as the above-mentioned copper uptake experiments. For this particular investigation, standard stock aqueous solutions (10^−3^ M) of nine different metal ions (copper included) were prepared, and then they were diluted to reach the working concentration of 250 µM. After analyzing the interaction of each individual metal with the matrix, the copper solution (250 µM) was mixed with each of the other eight metal solutions (250 µM), in a 1/1 volumetric ratio. The amount of copper and other metals was determined by SEM-EDAX. To do this, five different areas were scanned, and the results were expressed as average values.

##### Determination of Copper Ions from Solution

**Figure 10:**
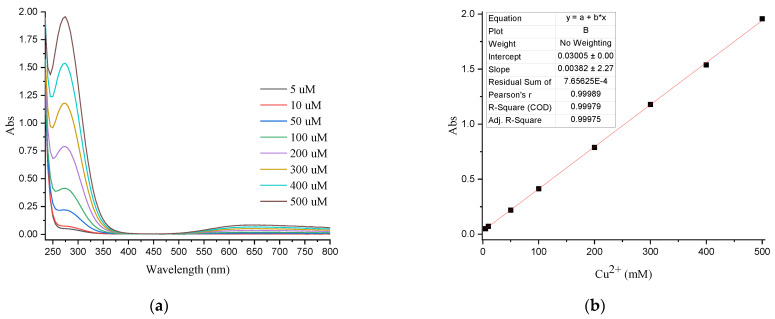
(**a**) UV-Vis spectra of the copper solutions (from 5 µM to 500 µM) in the presence of PEI (10^−2^ M); (**b**) Calibration curve (absorbance *vs* concentration plot (Cu^2+^: 5–500 µM).

The quantitative method used to determine the retention of copper ions was a spectrophotometric one, using polyethyleneimine as a chelating agent [57]. To do this, first, a calibration curve was generated, as follows. A 10^−3^ M stock aqueous solution of copper was prepared and then diluted to obtain eight solutions of different concentrations: 5, 10, 50, 100, 200, 300, 400, and 500 µM, respectively. An amount of 2.5 mL of each solution was mixed up with 100 µL PEI (10^−2^ M). This volume of the chelating agent was determined to be the optimum one, proved by a spectrophotometric titration using the most concentrated copper solution (500 µM). Further, the UV-Vis absorption spectra were recorded for each mixed solution, in the 200–800 nm range (Figure 10a). The calibration curve was plotted, using the absorption value at 275 nm, for each concentration in particular (Figure 10b).

In order to understand and to be aware of the limitation of this spectrophotometric method, we have made some calculations regarding the boundaries, namely the limit of detection (*LOD*) and limit of quantification (*LOQ*) [58,59]. Upon calculation, we established a *LOD* of 14 µM and a *LOQ* at around 44 µM, in terms of copper ion concentration.

For determination of the copper ions in solution, the solution to be analyzed was mixed with 100 µL PEI (10^−2^ M) and subjected to UV-vis spectrum recording, and the concentration was found by fitting the absorbance on the calibration curve.

##### Kinetics Studies

**Figure 11:**
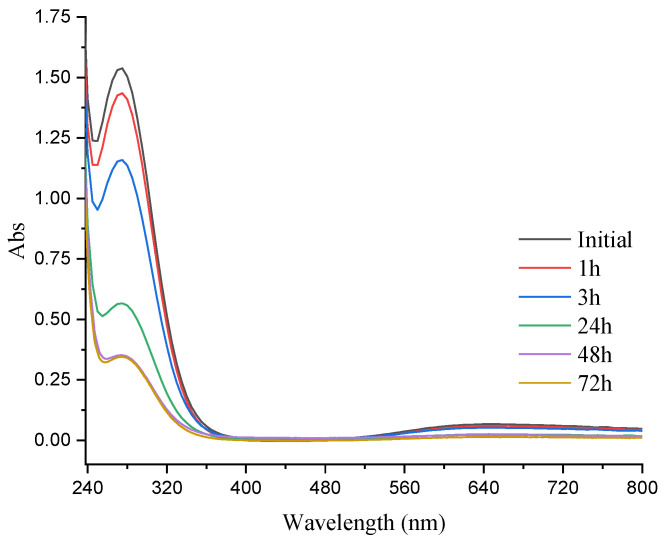
UV-Vis spectra of the copper solution (400 µM) after the immersion of the reference xerogel (X), at different times.

For the kinetic studies, in a copper solution of 400 µM a square piece of xerogel of known weight was immersed. From time to time (1; 3; 24; and 72 h), 2.5 mL of the supernatant was extracted and subjected to UV-Vis analysis (Figure 11), and the copper ion concentration was quantified using the already mentioned spectrophotometric method.

**Figure 12:**
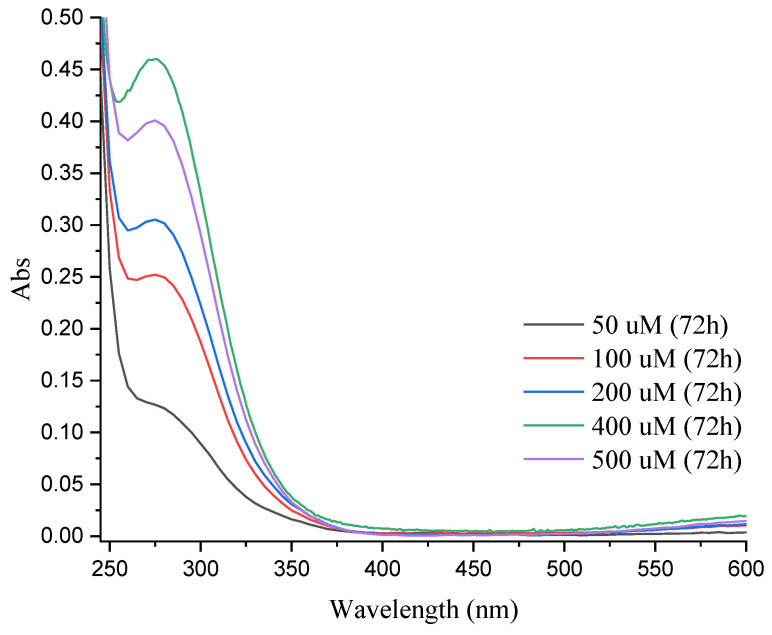
UV-Vis spectra of the copper aqueous solutions (50 to 500 µM) after 72 h interaction with the xerogel matrix.

In order to obtain the necessary experimental data for rendering and modeling adsorption isotherms, a batch adsorption assay followed by the UV-Vis analysis was utilized, involving five different copper solutions, with concentrations between 50 µM and 500 µM (Figure 12).

All experiments were performed in triplicate, and the average value was given.

##### The Adsorption Kinetics

The adsorption kinetics were investigated by fitting the in vitro data on three mathematical kinetic models, as follows:

*Pseudo-first-order*: q_t_ = q_e_(1 − e−^k1t^) (1)

*Pseudo-second-order*: q_t_ = (k2q_e_^2^t)/(1 + k2q_e_t) (2)

where: q_e_ is the amount of copper ions sorbed at equilibrium (mg/g); q_t_ is the amount of metal ion sorbed at time t (mg/g); and k1 and k2 are the rate constant of the pseudo-first-order kinetic model (min^−1^) and the pseudo-second-order kinetic model (g mg^−1^ min^−1^), respectively.

*The Weber–Morris intra-particle diffusion*: q_t_ = k_id_t^1/2^ + C_i_ (3)

where k_id_ is the intra-particle diffusion rate constant (g mg^−1^ min^−1/2^), and C_i_ is a constant related to the effect of boundary layer thickness.

The retention efficiency (R, %) was calculated using the following equation:

R = {(C_0_ − C)/C_0_}∗100 (4)

where: C_0_—the concentration of the metal ion in aqueous solution before the interaction with the xerogel (mg/L), and C—the concentration of the metal ion in aqueous solution (mg/L) after the sorption process.

##### Adsorption Isotherms

The experimental data, collected using the batch sorption technique, were examined by four isotherm models: Langmuir (linear model), Freundlich (nonlinear model), Sips (nonlinear model) and Dubinin–Radushkevich (nonlinear model) as well. The parameters investigated from the isotherm models were calculated using the following Equations (5)–(11):

(a) Langmuir model

1/q_e_ = 1/q_m_ + 1/q_m_kC_e_ (5)

R_L_ = 1/(1 + kC_i_) (6)

where: q_e_—the amount of copper ions sorbed per gram of material (mg/g); q_m_—theoretical maximum adsorption capacity (mg/g); k—Langmuir constant (L/mg); C_e_—equilibrium concentration of copper ions in the filtrate (mg/L); R_L_—constant separation factor; and C_i_—highest initial concentration in copper ions used in the experiment (mg/L).

(b) Freundlich model

q_e_ = k_F_C_e_^N^ (7)

where: k_F_—Freundlich constant, quantity of the metal ion per gram of material at equilibrium (mg/g); N—a parameter that describes the intensity of adsorption process; and q_e_ and C_e_ share the same meaning as in Equation (1).

(c) Sips model

q_e_ = q_m_kC_e_^N^/(1 + kC_e_^N^) (8)

where: q_e_, C_e_—same meaning as in Equation (1); q_m_—monolayer adsorption capacity (mg/g); and k—Sips constant.

(d) Dubinin–Radushkevich model

q_e_ = q_m_exp(−βε^2^) (9)

ε = RTln(1 + 1/C_e_) (10)

E = 1/(2β)^1/2^ (11)

where: q_e_—the amount of Cu^2+^ adsorbed (mg/g); q_m_—maximum adsorption capacity (mg/g); C_e_—equilibrium concentration of the metal ion (mg/L); β—Dubinin-Radushkevich constant (mol^2^/kJ^2^); ε—adsorption potential; R—gas constant (8.314 kJ/mol); T—absolute temperature (K); and E—free energy of adsorption process (kJ/mol).

## Figures and Tables

**Table 1:** Adsorption capacity of chitosan and its derivatives for copper ions.

Adsorbent	mg Cu^2+^/g Adsorbent	Reference
Chitosan	330	[17]
Epichlorohydrin crosslinked xanthate chitosan	43.47	[18]
Chitosan/poly(vinyl-alcohol) beads	47.9	[17]
Protonated chitosan beads	52	[19]
Carboxylated chitosan beads	86	[19]
Chitosan crosslinked with N-N^’^-[bis(2-hydroxy-3-formyl-5-methylbenzyldimethyl)]-ethylenediamine	113.6	[20]
Grafted chitosan beads	126	[19]
Ethylenediamine tetraacetic acid-grafted polyvinyl alcohol/chitosan beads	127.8	[21]
Chitosan/PVA-Fe_3_O_4_ magnetic composite	143	[22]
Chitosan crosslinked with 4-(2 pyridylazo)resorcinol (PAR)	170.23	[23]
Chitosan grafted with methyl metacrylate	192.3	[24]
EDTA-functionalized chitosan graphene oxide nanocomposites	207.26	[25]
Chitosan/(MCGON) graphene oxide nanocomposite	217.4	[26]
Lignosulfonate/chitosan material	319.2	[27]
Nanobentonite-cellulose/chitosan aerogel	1937.5	[28]
Phenothiazine-imine-chitosan material (CuX)	4394	This study

**Table 2:** Kinetic model parameters for Cu^2+^ sorption onto the reference xerogel (X).

**Parameter**	q_e_ (exp, mg/g)	**Pseudo-first-order model**					
		q_e_ (calc, mg/g)		k_1_		R^2^	χ^2^
CuX	3.7937	3.84342		0.0012		0.99767	0.00899
**Parameter**	q_e_ (exp, mg/g)	**Pseudo-second-order model**					
		q_e_ (calc, mg/g)		k_2_		R^2^	χ^2^
CuX	3.7937	4.61888		2.88993 × 10^−4^		0.9946	0.02085
**Parameter**	q_e_ (exp, mg/g)	**Intra-particle diffusion (Weber–Morris) model**					
		k_id1_	k_id2_	C_i1_	C_i2_	R^2^_1_	R^2^_2_
CuX	3.7937	0.09456	0.02608	0.47998	2.19302	0.99832	0.81258

**Table 3:** Isotherm parameters extracted from the experimental data plots.

Parameters					
Langmuir isotherm		q_m_ (mg/g)	K	R_L_	R^2^
		2.02416	0.14043	0.21756	0.87897
**Parameters**					
Freundlich isotherm		K_F_ (mg/g)	N	R^2^	χ^2^
		0.01451	3.32231	0.95466	0.34042
**Parameters**					
Sips isotherm	q_m_ (mg/g)	k	N	R^2^	χ^2^
	6.801	5.77 × 10^−7^	8.947	0.98785	0.13688
**Parameters**					
Dubinin–Radushkevich isotherm		q_m_ (mg/g)	β	E (kJ/mol)	R^2^
		0.9999	1.2939	0.62163	0.62202

## Data Availability

The data presented in this study are available on request from the corresponding author.
